# People react more positively to female- than to male-favoring sex differences: A direct replication of a counterintuitive finding

**DOI:** 10.1371/journal.pone.0266171

**Published:** 2022-03-30

**Authors:** Steve Stewart-Williams, Xiu Ling Wong, Chern Yi Marybeth Chang, Andrew G. Thomas

**Affiliations:** 1 School of Psychology, University of Nottingham Malaysia, Semenyih, Selangor Darul Ehsan, Malaysia; 2 School of Medicine, University of Nottingham, Nottingham, United Kingdom; 3 Department of Psychology, Swansea University, Swansea, United Kingdom; University of Padova, ITALY

## Abstract

We report a direct replication of our earlier study looking at how people react to research on sex differences depending on whether the research puts men or women in a better light. Three-hundred-and-three participants read a fictional popular-science article about fabricated research finding that women score higher on a desirable trait/lower on an undesirable one (female-favoring difference) or that men do (male-favoring difference). Consistent with our original study, both sexes reacted less positively to the male-favoring differences, with no difference between men and women in the strength of this effect. Also consistent with our original study, belief in male privilege and a left-leaning political orientation predicted less positive reactions to the male-favoring sex differences; neither variable, however, predicted reactions to the female-favoring sex differences (in the original study, male-privilege belief predicted positive reactions). As well as looking at how participants reacted to the research, we looked at their predictions about how the average man and woman would react. Consistent with our earlier results, participants of both sexes predicted that the average man and woman would exhibit considerable own-sex favoritism. In doing so, they exaggerated the magnitude of the average woman’s own-sex favoritism and predicted strong own-sex favoritism from the average man when in fact the average man exhibited modest other-sex favoritism. A greater awareness of people’s tendency to exaggerate own-sex bias could help to ameliorate conflict between the sexes.

## Introduction

### Male vs. female-favoring sex differences

Research on sex differences is sometimes controversial [1]. From the sociobiology wars of the 1970s to the scandals surrounding Lawrence Summers and James Damore, various culture-war flare-ups have centered on unwelcome claims about differences between men and women [2]. Some commentators point out, however, that not all sex-difference claims are equally likely to arouse concern or consternation [3]. One potentially important factor shaping people’s reactions to research on sex differences is whether the findings paint men or women in a better light. At first glance, there would appear to be three main predictions about how this would impact people’s reactions (assuming it impacts them at all).

1. *Anti-Female Bias*: People will react more positively to male-favoring findings.
2. *Pro-Female Bias*: People will react more positively to *female*-favoring findings.
3. *Gender Tribalism*: Men will react more positively to male-favoring findings; women will react more positively to female-favoring findings.

Existing theories in social psychology could in principle support each of these predictions. Traditional sexism might suggest a preference for male-favoring findings, either just in males or in females as well as a result of internalized misogyny [4]. Benevolent sexism and the chivalry hypothesis might suggest a preference for findings that favor females [4, 5]. And gender-ingroup bias might suggest gender tribalism: a male preference for male-favoring findings and a female preference for female-favoring findings [6–10].

Because existing theories can predict all possible outcomes, they give us little guidance in framing predictions in this domain. To break the theoretical gridlock, we proposed a new theory grounded in universal physical sex differences, which we dubbed the *greater-protectiveness-of-females* (or *G-PROF*) theory. The central claim of the G-PROF theory is that, as a general rule, people are more protective and/or controlling of girls and women than of boys and men. This, we argue, traces back ultimately to certain physical and reproductive differences between the sexes. First, men are typically larger and stronger than women, which means that women are typically more vulnerable. Second, women rather than men get pregnant and nurse the young, which means that, if a woman gets pregnant without an investing partner, she and her family are likely to be left “holding the baby.” In both cases, these sex differences may push individuals toward greater protectiveness and/or control of females than males—tendencies that may eventually be enshrined in the norms of a culture. A third sex difference may lead to the same destination but via a different route: cultural group selection [11]. The sex difference in question is the fact that women are more “reproductively indispensable”: If half the men in a group were removed, the group could in principle produce as many offspring in the next generation; if half the *women* were removed, the group could not. That being the case, groups that were more protective and controlling of women may have survived longer and grown more rapidly, and those tendencies would have slowly proliferated [12].

It is worth emphasizing that, although this explanation is grounded in evolved differences between the sexes, it is first-and-foremost a sociocultural explanation: Our primary claim is that these evolved differences influence learned behavior and social norms. The reason for this emphasis is that the extent to which girls and women are protected varies a great deal across cultures, and this is easier to reconcile with a sociocultural than an evolutionary explanation. That said, it does seem possible that the physical and reproductive sex differences that sometimes push individuals and cultures to be more protective and/or controlling of females could also have helped shape an evolved inclination in the same direction. Thus, the tendency may have both sociocultural and evolutionary roots.

In any case, applying the G-PROF theory to the question of reactions to research on sex differences led us to predict that people would typically react more positively to findings that put women rather than men in a more favorable light—that is, Option 2 on the earlier list. In a pre-registered study, we presented participants with fictitious popular-science articles describing sex differences that either favored females (females draw better or lie less) or favored males (the opposite). As expected, participants of both sexes were more positively disposed toward the female- than the male-favoring findings, judging the former to be more important, and the latter to be more harmful, offensive, and upsetting. We had predicted that, although the pro-female tendency would be found in both sexes, it would be stronger in women than men due to gender-ingroup bias [6–10]. Contrary to expectations, however, there was little evidence for such an effect: The preference for female-favoring differences was comparable in magnitude for both sexes. A follow-up study in Southeast Asia replicated both the male-favoring aversion and the absence of a sex difference, suggesting that this pattern extends beyond the boundaries of the West.

Several variables moderated participants’ reactions to the research. The first was belief in male privilege. The more privileged that participants believed men are over women, the more negatively they reacted to the male-favoring differences and the more positively to the female-favoring ones (and vice versa for the minority of participants who believed that women are privileged over men). The second moderator was political orientation: Independently of male-privilege belief, the more that participants leaned politically to the left, the more negatively they reacted to the male-favoring differences. Political orientation did not predict reactions to the female-favoring differences, perhaps because the protection of females is not a consideration when females are doing better. In our Southeast Asian replication, male-privilege belief predicted negative reactions to the male-favoring differences but did not predict reactions to the female-favoring ones, and political orientation did not predict reactions at all.

### Lay predictions about gender bias

An additional issue explored in our earlier study was people’s beliefs about men and women’s gender-ingroup biases. Specifically, we explored participants’ predictions about how the average man and woman would respond to the male- vs. female-favoring sex differences. Our expectation was that both sexes would greatly overestimate the level of ingroup bias in both their own sex and the other. This was based on the fact that people tend to overestimate the magnitude of *most* effects in psychology [e.g., 13], coupled with the fact that people may have a heightened sensitivity to any evidence that individuals or groups are acting in self-interested ways, as the costs of overlooking such information are likely to be higher than the costs of an occasional false alarm [cf: 14]. As predicted, and consistent with past research [15], participants of both sexes predicted that the average man would strongly prefer the male-favoring findings, whereas the average woman would strongly prefer the female-favoring ones. The participants’ predictions were erroneous: First, participants greatly exaggerated the magnitude of the average woman’s own-sex preference, and second, participants assumed that the average man would exhibit a strong own-sex preference when in fact he exhibited a modest other-sex preference. These results were fully replicated in our Southeast Asian sample, suggesting again that the phenomenon is not simply a WEIRD or a Western one.

### Aims and hypotheses

The aversion to male-favoring sex differences is contrary to what many would expect and could have significant theoretical and practical implications—shedding light, for instance, on the conduct and reception of research on sex differences, challenging common views about gender stereotypes and gender-ingroup bias, and helping quell conflict between the sexes. As such, it is important to assess the replicability of the finding. That was the aim of the present study. More precisely, the aim was to conduct a direct replication of our initial study on the topic [2], with a comparable Western sample and the same materials. The hypotheses below are based on the results of the earlier study, which was pre-registered with OSF (https://osf.io/6n5up/).

1. Participants will react more positively to female- than male-favoring sex differences.
2. Participant sex will have no impact on the strength of this effect.
3. The more privileged that participants think men are over women, the more negatively they will react to male-favoring differences and the more positively to female-favoring differences.
4. The more that participants lean to the left politically, the more negatively they will react to male-favoring differences. Political orientation will not predict reactions to female-favoring differences.
5. Both sexes will greatly overestimate the extent to which the average man and woman exhibit a preference for own-sex-favoring sex differences.

Note that Hypotheses 1 and 5 are the same as the original study, as the results were consistent with our hypotheses; Hypothesis 2 differs from the original study (we initially predicted that the preference for female-favoring findings would be stronger for female participants, but did not find this); and Hypotheses 3 and 4 were not part of the original study (the patterns emerged through exploratory analysis).

## Methods

The study reported here was approved in full by the Science and Engineering Research Ethics Committee (SEREC) of the University of Nottingham Malaysia (Approval Number: SSW280818). Consent was recorded via an online form; participants were presented with some background information about the study followed by a consent statement: “I have read and understood the information above, and consent to take part in this study.” Contingent on an affirmative response, they were then directed to the main survey.

### Participants

Participants were recruited via Prolific.ac, a website offering people a small payment for participating in online studies. We calculated the minimum sample size in advance with G*Power 3.1 [16], using an a priori power test (“ANOVA: Fixed effects, main effects, and interactions”) for three independent variables: Sex Favored (female vs. male), Participant Sex (female vs. male), and Trait Valence (positive vs. negative). Alpha was set at .05, power at .95, and the minimum effect size of interest (*f*) at 0.25 (a medium effect, which was the most common effect size found in our initial study). The analysis produced a recommended minimum sample size of 210. Our final sample consisted of 303 individuals: 150 men and 153 women. The age range was 18 to 72 years (*M* = 34.16, *SD* = 11.43). Most participants were from either the United States (64.4%) or the United Kingdom (32.3%).

### Materials and procedure

The study was run with the online survey platform Qualtrics. After providing some general biographical information, participants were presented with a fictitious popular-science article discussing a recent study finding a sex difference (also fictitious; see S1 Fig for a sample). For half the participants, this was a sex difference in a positive trait (drawing ability); for the other half, it was a sex difference in a negative trait (lying frequency). Within each condition, half the participants were shown a female-favoring difference, and half were shown a male-favoring difference. Each version of the article included both a discussion of the sex difference and a bar graph representing it. Having read the article, participants answered four short questionnaires.

#### Reaction-to-Research questionnaire

This questionnaire asked participants about their reactions to the sex-difference study and its supposed findings. More precisely, it asked them how interesting, important, plausible, surprising, offensive, harmful, and upsetting the findings were, how well conducted the research was, and how sexist research of this kind is. Responses were registered on seven-point Likert scales, with anchors appropriate to each item (e.g., “Not very plausible at all” vs. “Extremely plausible”).

#### Average-man and average-woman predictions questionnaires

The next two questionnaires asked participants how they thought the average man and woman participating in the study would react to the research. This involved giving them the Reaction-to-Research questions again twice and asking them, first, to respond in the way they thought the average man would, and second, to respond in the way they thought the average woman would.

#### Male-privilege belief scale

Finally, participants completed a four-item Male-Privilege Belief Scale, based on Martin and Nezlek’s Belief in White Privilege Scale [17]. The modified scale asked participants for their views about how privileged men and women are relative to one another in society. One item, for example, asked “Do you think men have fewer advantages or more advantages in life than women?” Responses were again registered on 7-point Likert scales with options appropriate to each item (e.g., “Men have many fewer advantages” at one end of the scale vs. “Men have many more advantages” at the other).

Having completed the survey, participants were informed that the popular-science article, and the research it described, were fictitious. Note that we are reporting all the measures and manipulations used in this study.

### Statistical analyses

All analyses were conducted with SPSS Version 26. No outliers were identified or removed; all Cook’s Distance values were < 1. Four aggregate variables were created from the individual items in the four questionnaires: participants’ reaction to the research, participants’ predictions about the average man’s reaction, participants’ predictions about the average woman’s reaction, and belief in male privilege. For the three reaction questionnaires, we reverse-scored the items indicating a negative reaction to the research (i.e., those assessing how surprising, offensive, harmful, upsetting, and inherently sexist it was). As such, higher scores on these variables indicated a more positive reaction. All four variables had good internal consistency (Reaction to Research: α = .82; Average Man Predicted: α = .88; Average Woman Predicted: α = .9; Male-Privilege Belief Scale: α = .9).

To examine the effects of Sex Favored and Participant Sex on reactions to the research, and on predictions about the reactions of the average man and woman, we ran a series of two-way ANOVAs. As with the original study, we did not include Trait Valence as a third independent variable, as preliminary analyses showed that there was no main effect of this variable and no interactions between it and the other independent variables (main effect of Trait Valence: *F*_1, 295_ = 0.12, *p* = .729, *d* = 0.03; interaction with Sex Favored: *F*_1, 295_ = 0.21, *p* = .644, *d* = 0.06; interaction with Participant Sex: *F*_1, 295_ = 0.00, *p* = .986, *d* = 0.01; interaction with Sex Favored *and* Participant Sex: *F*_1, 295_ = 1.69, *p* = .194, *d* = 0.15). To determine whether male-privilege belief and political orientation moderated the effect of Sex Favored on participants’ reactions to the research, we conducted a moderation analysis using PROCESS Model 2 [18]. All variables were mean-centered before running the analysis [19, 20]. Interactions were explored using post-hoc multiple regressions. Our data met all the assumptions for multiple regression, including normality, linearity, homoscedasticity, and absence of multicollinearity. The minimal dataset is freely available from OSF at https://osf.io/ctnez/.

The threshold for statistical significance for all analyses was set at *p* = .05. We did not correct for multiple comparisons as we had specific predictions for each of our dependent variables and are reporting all our results. See S1-S5 Tables in S1 File for a breakdown of the descriptive and inferential statistics pertaining to this study.

## Results

### Sex favored and participant sex

**Fig 1:**
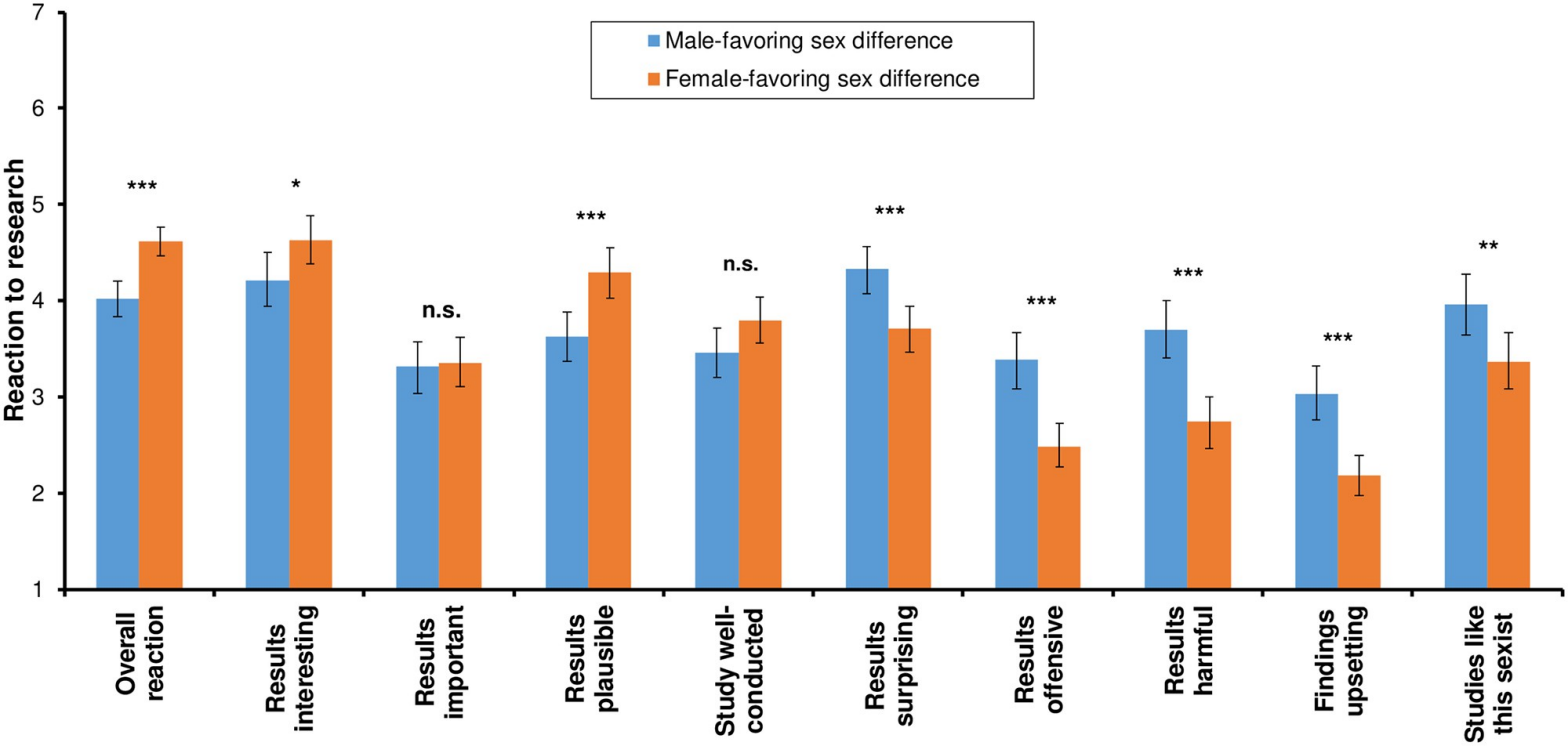
Participants’ reactions to fictitious research describing a male- vs. a female-favoring sex difference. Higher scores on the “Overall reaction” variable indicate a more positive reaction to the research; higher scores on the individual items indicate stronger agreement that the descriptor applies to the research. * *p* < .05; ** *p* < .01; *** *p* < .001; n.s.: not significant. Error bars = 95% CIs.

We first examined participants’ overall reactions to the fictitious research. Consistent with Hypothesis 1, there was a main effect of Sex Favored: Participants reacted less positively to the male-favoring sex differences than the female-favoring ones (*F*_1, 299_ = 24.48, *p* < .001, *d* = 0.58; see Fig 1, leftmost bars). As with our initial study, there was also a main effect of Participant Sex: Collapsing across experimental conditions, men were somewhat more positive about the research (*F*_1, 299_ = 7.12, *p* = .008, *d* = 0.33). Finally, and consistent with Hypothesis 2, there was no interaction between Participant Sex and Sex Favored, indicating that male and female participants preferred the female-favoring over the male-favoring sex differences to a comparable degree (*F*_1, 299_ = 1, *p* = .318, *d* = 0.12).

To get a more detailed picture of the participants’ reactions, we also examined the individual items making up the aggregate variable. Fig 1 presents the results. Mirroring our original study, participants found the female-favoring sex differences more plausible, and the male-favoring differences more surprising, harmful, upsetting, and sexist. In contrast to the original study, participants also found the female-favoring sex differences more interesting but did not rate them as more important or the research as better conducted.

### Male-privilege belief and political orientation

The average participant viewed men as more privileged than women (*M* = 1.19, *SD* = 1.25, on a scale ranging from -3 to 3, with positive values indicating a belief that men are privileged over women and negative values the reverse). On average, though, female participants thought the privilege gender gap was larger than did males (*M* = 1.55, *SD* = 1.09 vs. *M* = 0.82, *SD* = 1.25; *F*_1, 299_ = 29.46, *p* < .001, *d* = 0.63). A minority of participants (12.9%) saw women as more privileged than men. This included 22.1% of the males and 4.8% of the females (*X*^*2*^ [1, *N* = 278] = 18.55, *p* < .001, *d* = 0.53).

Political orientation was measured with a Likert scale spanning from 1 (“Left/Liberal”) to 7 (“Right/Conservative”). Participants fell across the full range of the political spectrum, but with the average falling somewhat to the left (*M* = 3.26, *SD* = 1.58, where 4 indicates the centrist midpoint).

To test Hypotheses 3 and 4, we next looked at whether male-privilege belief and political orientation moderated the effect of Sex Favored on participants’ reactions to the research. The overall model was significant (R^2^ = .19, *F*_5_,_285_ = 13.66, *p* < .001). Sex Favored was the strongest predictor of participants’ overall reactions (*B* = 0.57, *t*_285_ = 4.97, *p* < .001), followed by male-privilege belief (*B* = -0.13, *t*_285_ = -2.31, *p* = .022). Political orientation did not uniquely predict participants’ reactions (*B* = 0.08, *t*_285_ = 1.93, *p* = .054).

There was a significant interaction between Sex Favored and male-privilege belief (*B* = 0.38, *t*_285_ = 3.49, *p* = .001). Consistent with Hypothesis 3, the more privileged that participants believed men are over women, the less positively they reacted to the male-favoring sex differences (R^2^ = .18, *F*_1, 148_ = 32.79, *p* < .001, *B* = -0.39, *t*_148_ = -5.73, *p* < .001). Contrary to Hypothesis 3, though, male-privilege belief did not predict reactions to the female-favoring differences (R^2^ = .002, *F*_1, 149_ = 0.36, *p* = .548, *B* = 0.04, *t*_149_ = 0.6, *p* = .548).

There was no interaction between Sex Favored and political orientation, contrary to Hypothesis 4 (*B* = -0.1, *t*_285_ = -1.23, *p* = .221). However, a post-hoc analysis using PROCESS Model 1 revealed that, without including male-privilege belief in the analysis, there *was* a significant Sex Favored × Political Orientation interaction (R^2^ = .15, *F*_3, 289_ = 16.41, *p* < .001, *B* = -0.26, *t*_289_ = -3.45, *p* = .001): Consistent with Hypothesis 4, a left-leaning political orientation predicted more negative reactions to male-favoring differences (*R*^2^ = .21, *F*_2, 144_ = 19.5, *p* < .001, *B* = 0.13, *t*_144_ = 2.23, *p* = .028), but did not predict reactions to the female-favoring differences (*R*^2^ = .006, *F*_2, 141_ = 0.41, *p* = .663, *B* = 0.03, *t*_141_ = 0.49, *p* = .623). The fact that, after adding male-privilege to the model, the Sex Favored × Political Orientation interaction disappeared indicates that, in this dataset at least, much of the link between a left-leaning political orientation and negative reactions to male-favoring sex differences is a result of the fact that left-leaning individuals typically perceive higher levels of male privilege.

### Predictions about the average man and woman’s gender biases

**Fig 2:**
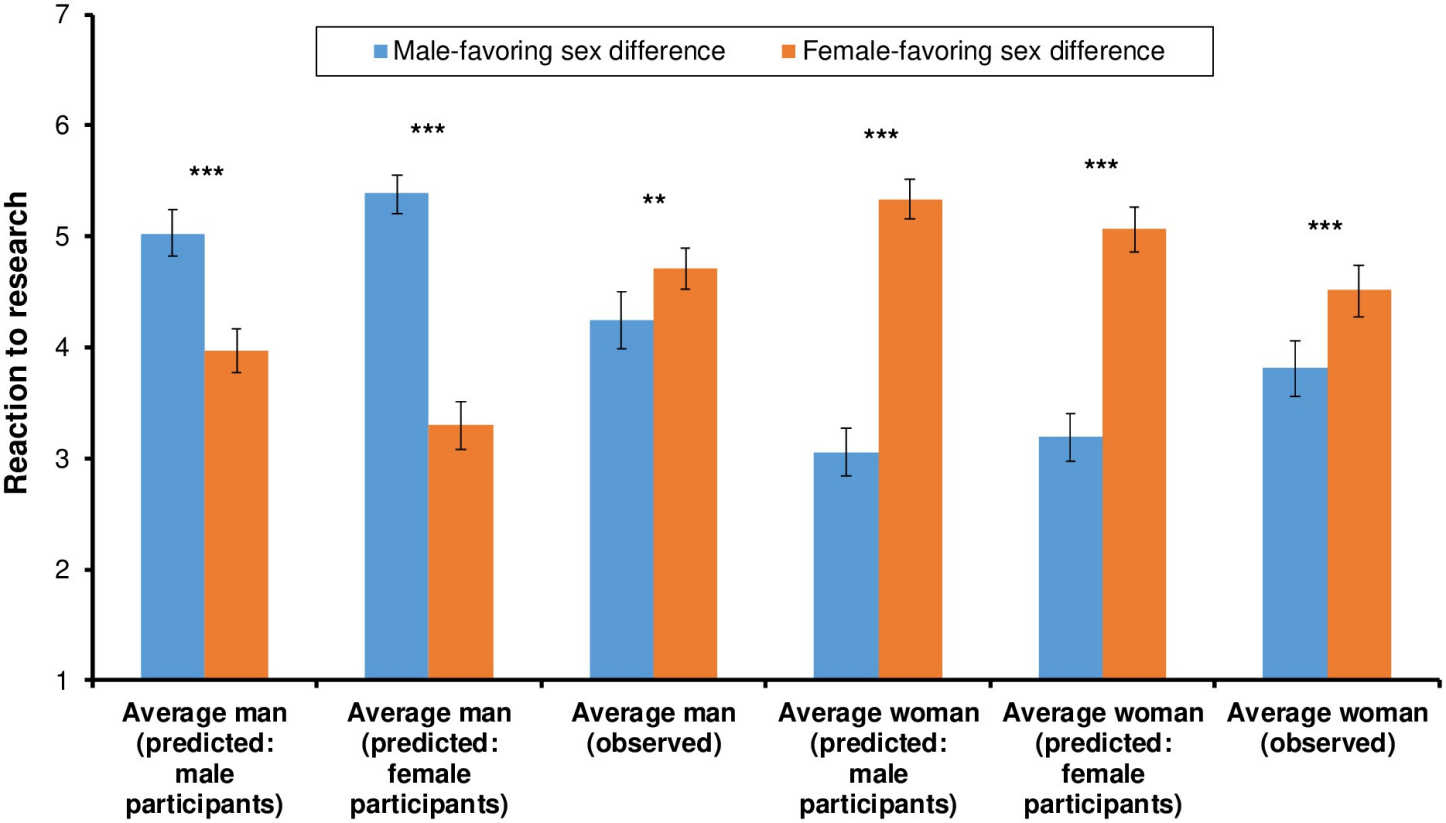
Participants’ predictions regarding the reactions of the average man and woman to a male- vs. female-favoring sex difference, alongside the actual reactions observed in the study. * *p* < .05; ** *p* < .01; *** *p* < .001; n.s.: not significant. Error bars = 95% CIs.

Finally, we looked at participants’ predictions regarding the reactions of the average man and woman to the fictitious findings. Starting with the average man, there was a large main effect of Sex Favored: Consistent with Hypothesis 5, participants predicted that the average man would react much more positively to the male- than the female-favoring sex differences (*F*_1, 298_ = 225.02, *p* < .001, *d* = 1.66; see Fig 2). The collective prediction was false: As discussed, the average man in fact reacted less positively to the *male*-favoring differences. There was no main effect of Participant Sex (*F*_1, 298_ = 2.11, *p* = .148, *d* = 0.05). As with our earlier study, however, there was a significant Sex Favored × Participant Sex interaction (*F*_1, 298_ = 24.57, *p* < .001, *d* = 0.57): Female participants predicted more own-sex favoritism from the average man than male participant did. More precisely, female participants predicted that the average man would react more positively to the male-favoring differences than male participants predicted (*F*_1, 148_ = 7.18, *p* = .008, *d* = 0.44), and less positively to the female-favoring differences (*F*_1, 150_ = 18.02, *p* < .001, *d* = 0.69).

Turning to the average-woman predictions, there was an extremely large effect of Sex Favored on participants’ reactions to the research (*F*_1, 297_ = 426.38, *p* < .001, *d* = 2.38). Consistent with Hypothesis 5, participants predicted that the average woman would react much more positively to the female- than the male-favoring sex differences (*F*_1, 297_ = 426.38, *p* < .001, *d* = 2.38; see Fig 2). Though accurate with respect to its direction, the predicted effect was 361% larger than the actual one (*d* = 2.38 vs. 0.66). There was no main effect of Participant Sex (*F*_1, 297_ = 0.44, *p* = .509, *d* = 0.14). However, unlike our earlier study, there was a (barely) significant interaction between Sex Favored and Participant Sex (*F*_1, 297_ = 3.94, *p* = .048, *d* = 0.23): Males predicted slightly more own-sex bias from the average woman than females predicted—the mirror image of the pattern for the average man. This was largely because males predicted that the average woman would react more positively to the female-favoring differences than females predicted (*F*_1, 149_ = 3.9, *p* = .05, *d* = 0.32). The sexes did not differ in their predictions for the male-favoring differences (*F*_1, 148_ = 0.79, *p* = .375, *d* = 0.15).

## Discussion

The present study replicated most of the results of our original one [2]. As expected, participants of both sexes reacted less positively to research finding a male- than a female-favoring sex difference, consistent with the idea that people tend to be more protective of females than males. Contrary to our original expectation, but consistent with our earlier study, the male-favoring aversion was no stronger for our female participants than our male ones. This suggests that, at least in this arena, any effects of gender-ingroup bias are considerably weaker than the tendency to react less positively to male- than to female-favoring findings.

The more privileged that participants believed men are over women, and the more that participants leaned politically to the left, the less positively they reacted to the male-favoring differences. At the same time, neither political orientation nor male-privilege belief predicted reactions to the female-favoring differences. Although we did not anticipate this pattern, it does fit with the idea that people are more protective of women than men. People who think that men are greatly privileged over women, and who lean to the left politically, are more likely to see women as downtrodden and as victims of male misbehavior [2, 21]. As such, they are more likely to be protective of females in any circumstance where protectiveness might be called for. A study revealing a male-favoring sex difference is potentially such a circumstance, and thus male-privilege belief and political orientation may predict people’s reactions to such a study. In contrast, a study revealing a *female*-favoring sex difference would not call for protectiveness, regardless of one’s beliefs about male privilege or one’s political commitments, and thus these variables would not be expected to predict people’s reactions. The overall pattern of results is therefore consistent with the idea that the protectiveness of women underpins the widespread aversion to male-favoring findings.

Participants of both sexes predicted that the average man and woman would exhibit substantial own-sex favoritism. In making these predictions, they exaggerated the average woman’s own-sex favoritism and incorrectly assumed own-sex favoritism on the part of the average man. One could argue that the predictions regarding the average woman are an instance of *stereotype accuracy* [22]; after all, the average woman did indeed exhibit own-sex favoritism. However, given that participants’ predictions about the magnitude of this favoritism were so far off the mark, one could just as well argue the reverse: that when it comes to gender-ingroup bias, people’s stereotypes are systematically misaligned with reality.

Like the original study, female participants predicted more own-sex favoritism from the average man than did males. Unlike the original study, the reverse was true as well: Male participants predicted slightly more own-sex favoritism from the average woman than did females. Ironically, then, participants of both sexes exhibited own-sex favoritism in their predictions about own-sex favoritism.

### Limitations and future directions

The present study had a number of limitations. One is that our fictitious popular-science articles omitted a piece of information often included in such articles and likely to impact people’s reactions to sex-difference research: the sex of the researcher producing the research. If, as we suspect, the aversion to male-favoring findings stems from greater protectiveness of females than males, then the effect may be compounded when the research is produced by a male. Historically, men have been more of a threat to women than other women have, and thus the female-protective tendency may be intensified when a perceived threat originates from a man. If this reasoning is correct, then people may judge a male-favoring finding promoted by a male in a dimmer light than they would the same finding promoted by a female.

A second limitation is that we looked at the effects of male- vs. female-favoring sex differences for only two traits: drawing ability and lying frequency. To assess the generality of the male-favoring aversion, the range of traits should be extended. One trait that may be particularly worth exploring is intelligence. Intelligence is a reliable predictor of many important life outcomes, including academic achievement, career success, income, health, and longevity [23]. As such, sex differences in intelligence are more consequential and more contentious than those explored in our studies to date. Would the effect of Sex Favored be amplified for such a difference? Would men still exhibit an other-sex bias, or would they instead start exhibiting an own-sex bias given the higher psychological stakes? Based on the robustness of the male-favoring aversion in our studies so far, and the fact that the phenomenon that led us to predict it (i.e., the greater protectiveness of women) would if anything be *more* relevant for a consequential trait than an innocuous one, our expectation is that participants of both sexes would continue to react less positively to a male-favoring difference, but that the effect would be larger than that found in our prior research. This remains, however, to be demonstrated.

A final limitation of the present study is that we did not directly explore the psychological variables underpinning the aversion to male-favoring findings. One way to do so would be to split the Reaction-to-Research variable into three parts, namely the quality of the research, the harmfulness of its findings, and the extent to which the disfavored sex should be shielded from those findings. This would enable us to address two new questions. First, we could determine whether Sex Favored has an impact on judgments of research quality alone. Unlike judgments about harm and the need to shield the disfavored sex, judgments about quality should in principle be unaffected by the direction of the effect. As such, exploring the effect of Sex Favored on quality judgments alone would provide a stronger test of the effects of motivated reasoning on participants’ reactions. Second, if Sex Favored does indeed affect quality judgments (as we suspect it will), we could then explore whether this effect is mediated by participants’ perception of the harmfulness of the research findings and their desire to shield women from them.

## Conclusion

The present study replicated almost all the findings of our original one, using a comparable sample and identical materials. Participants of both sexes reacted less positively to male- than to female-favoring sex differences, and did so to the same extent. Male-privilege belief and a left-leaning political orientation predicted negative reactions to male-favoring differences but did not predict reactions to female-favoring ones. Although participants of both sexes reacted less positively to the male-favoring differences, participants *predicted* that both sexes would react much more positively to sex differences favoring their own sex. These predictions were mistaken: Female participants exhibited only a modest own-sex-favoring preference, and male participants exhibited a modest preference favoring the other sex. By challenging the common belief that both sexes are strongly biased in favor of their own sex, our findings may help quell some of the antagonism between the sexes seen in modern culture-war battles over gender issues.

## Supporting information

S1 Fig Fictitious popular-science article. (PDF)

S1 File Descriptive and inferential statistics for the paper “People react more positively to female- than to male-favoring sex differences: A direct replication of a counterintuitive finding”. (PDF)
