# Supplementary material for: People react more positively to female- than to male-favoring sex differences: A direct replication of a counterintuitive finding
Source: PLoS One. 2022 Mar 30;17(3):e0266171. doi: 10.1371/journal.pone.0266171 (PMC8967052; doi:10.1371/journal.pone.0266171)
Supplement: S1 Fig — (PDF) [file pone.0266171.s001.pdf]

Positive Trait Female-Favouring Difference

Sex Differences in Drawing Ability

Scientists recently conducted a large study looking at sex differences in drawing ability.

Below is an excerpt from a popular science article summarizing the findings of the study. **Please read the excerpt and study the graph carefully - carefully enough that you'll be able to answer questions about them later.**

DRAWING CONCLUSIONS: ARE MEN OR WOMEN BETTER AT DRAWING?

A new battleground has opened up in the war of the sexes: Are men better at drawing or are women? You might have your own opinion about this, but recently a group of scientists set out to answer the question more rigorously. They did five experiments in 19 countries, and their results were crystal clear: Although there are exceptions, in general *women's drawings are better*. They're more accurate, more creative, and just more pleasant to look at. Sorry men!

Discussing the findings, the lead researcher said: “We were surprised at just how consistent the effect was. In every country we looked at, women were better at drawing. The difference wasn’t always large, but it was always there. Now we just need to work out why!”

So next time the question comes up at a dinner party or in class, you’ll know the answer: Women draw better than men!

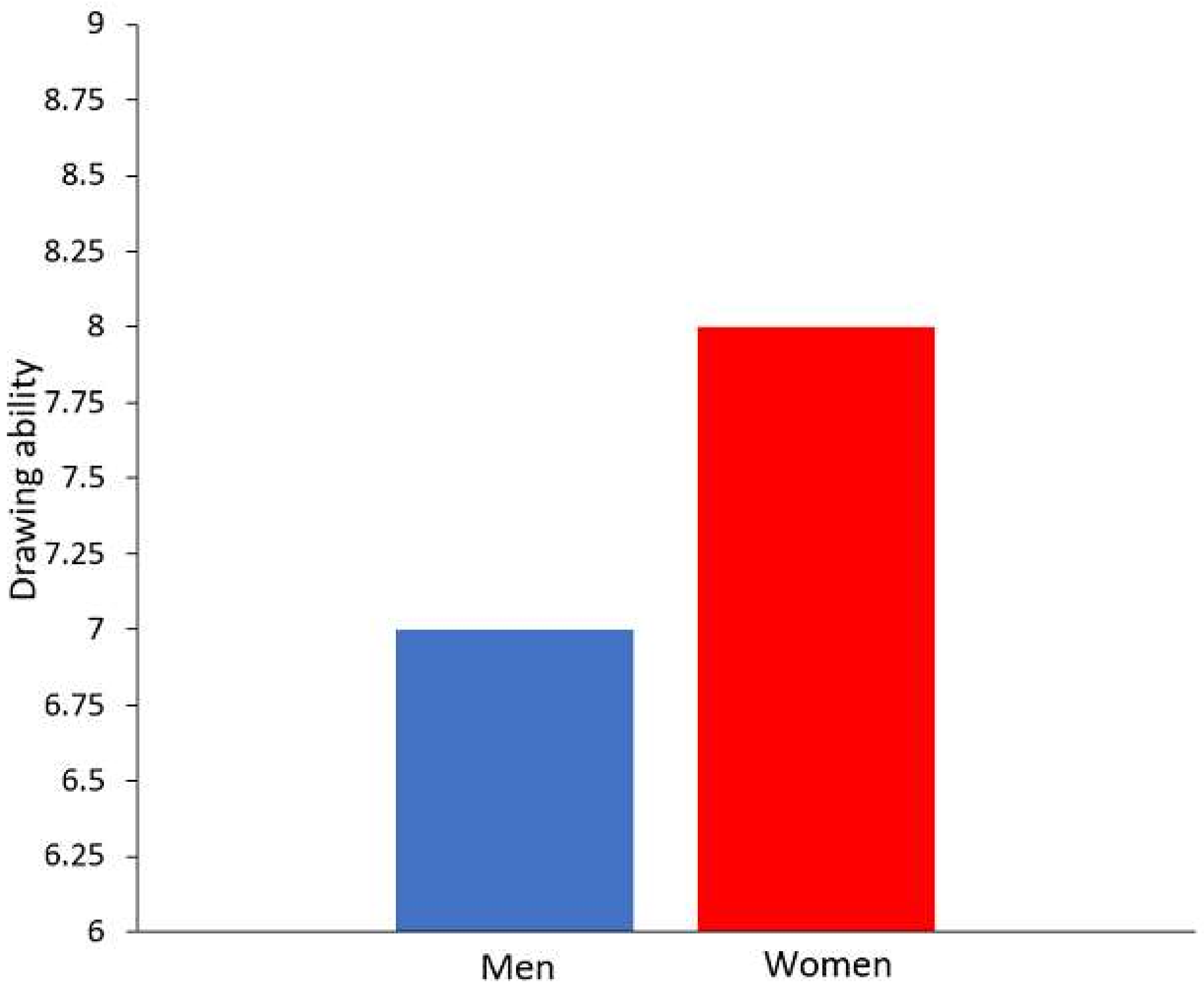

Figure 1: Sex differences in drawing ability. Across cultures, women score higher in drawing ability than men, on average.

When you're confident you understand the excerpt and the graph well enough to answer questions about them, click on the arrow below to move to the next section.

Powered by Qualtrics
