# Supplementary material for: People react more positively to female- than to male-favoring sex differences: A direct replication of a counterintuitive finding
Source: PLoS One. 2022 Mar 30;17(3):e0266171. doi: 10.1371/journal.pone.0266171 (PMC8967052; doi:10.1371/journal.pone.0266171)
Supplement: S1 File — (PDF) [file pone.0266171.s002.pdf]

S1-S5 Tables

Descriptive and inferential statistics for the paper “People react more positively to female- than to male-favoring sex differences: A direct replication of a counterintuitive finding.”

S1 Table. Participants’ reactions to the hypothetical sex-differences research by sex favored.

	**Sex Favored**					
	Male		Female		Total	
**Participant Sex**	*M*	*SD*	*M*	*SD*	*M*	*SD*
Combined	4.02	1.12	4.62	0.93	4.32	1.07
Male	4.25	1.09	4.71	0.86	4.50	1.00
Female	3.82	1.11	4.52	1.00	4.15	1.11

S2 Table. Inferential statistics related to participants’ reactions to the hypothetical sex-differences research by sex favored.

	df	*F*	*p*	*d*
Sex Favored	1, 299	24.48	.000	0.58
Participant Sex	1, 299	7.12	.008	0.33
Sex Favored * Participant Sex	1, 299	1	.318	0.12

S3 Table. Participants' reaction to research by sex favored: Individual items.

	Sex Favored							
	Male		Female					
**Item**	*M*	*SD*	*M*	*SD*	df	*F*	*p*	*d*
Results interesting	4.21	1.76	4.63	1.54	1, 299	5.15	.024	0.25
Results important	3.31	1.75	3.35	1.54	1, 295	0.07	.799	0.03
Results plausible	3.63	1.67	4.29	1.60	1, 296	11.26	.001	0.40
Study well-conducted	3.46	1.67	3.80	1.51	1, 299	3.13	.078	0.21
Results surprising	4.33	1.53	3.71	1.51	1, 292	11.79	.001	0.41
Results offensive	3.39	1.76	2.48	1.47	1, 296	21.81	.000	0.56
Results harmful	3.69	1.82	2.74	1.65	1, 295	21.40	.000	0.55
Findings upsetting	3.02	1.72	2.18	1.34	1, 295	20.69	.000	0.55
Studies like this inherently sexist	3.96	1.92	3.37	1.79	1, 299	6.71	.010	0.32

S4 Table. Participants' predictions about how the average man and average woman's overall reactions to the research.

		**Sex Favored**					
		Male		Female		Total	
	**Participant Sex**	*M*	*SD*	*M*	*SD*	*M*	*SD*
**Average Man (Predicted)**	Combined	5.22	0.86	3.65	1.03	4.43	1.23
	Male	5.02	0.85	3.97	1.00	4.46	1.07
	Female	5.39	0.83	3.30	0.94	4.40	1.37
**Average Woman (Predicted)**	Combined	3.13	0.91	5.20	0.83	4.17	1.36
	Male	3.06	0.89	5.33	0.77	4.26	1.41
	Female	3.19	0.93	5.06	0.88	4.08	1.30

S5 Table. Inferential statistics related to participants' predictions about how the average man and average woman's overall reactions to the research.

		df	*F*	*p*	*d*
**Average Man (Predicted)**	Sex Favored	1, 298	225.02	.000	1.66
	Participant Sex	1, 298	2.11	.148	0.05
	Sex Favored * Participant Sex	1, 298	24.57	.000	0.57
**Average Woman (Predicted)**	Sex Favored	1, 297	426.38	.000	2.38
	Participant Sex	1, 297	0.44	.509	0.14
	Sex Favored * Participant Sex	1, 297	3.94	.048	0.23
